# A content-based dataset recommendation system for researchers—a case study on Gene Expression Omnibus (GEO) repository

**DOI:** 10.1093/database/baaa064

**Published:** 2020-11-12

**Authors:** Braja Gopal Patra, Kirk Roberts, Hulin Wu

**Affiliations:** Department of Biostatistics and Data Science, School of Public Health, The University of Texas Health Science Center at Houston/1200 Pressler Street, Suite E-833, Houston, TX, 77030, USA and; School of Biomedical Informatics, The University of Texas Health Science Center at Houston/7000 Fannin st. Suite 600, Houston, TX, 77030, USA; Department of Biostatistics and Data Science, School of Public Health, The University of Texas Health Science Center at Houston/1200 Pressler Street, Suite E-833, Houston, TX, 77030, USA; School of Biomedical Informatics, The University of Texas Health Science Center at Houston/7000 Fannin st. Suite 600, Houston, TX, 77030, USA

## Abstract

It is a growing trend among researchers to make their data publicly available for experimental reproducibility and data reusability. Sharing data with fellow researchers helps in increasing the visibility of the work. On the other hand, there are researchers who are inhibited by the lack of data resources. To overcome this challenge, many repositories and knowledge bases have been established to date to ease data sharing. Further, in the past two decades, there has been an exponential increase in the number of datasets added to these dataset repositories. However, most of these repositories are domain-specific, and none of them can recommend datasets to researchers/users. Naturally, it is challenging for a researcher to keep track of all the relevant repositories for potential use. Thus, a dataset recommender system that recommends datasets to a researcher based on previous publications can enhance their productivity and expedite further research. This work adopts an information retrieval (IR) paradigm for dataset recommendation. We hypothesize that two fundamental differences exist between dataset recommendation and PubMed-style biomedical IR beyond the corpus. First, instead of keywords, the query is the researcher, embodied by his or her publications. Second, to filter the relevant datasets from non-relevant ones, researchers are better represented by a set of interests, as opposed to the entire body of their research. This second approach is implemented using a non-parametric clustering technique. These clusters are used to recommend datasets for each researcher using the cosine similarity between the vector representations of publication clusters and datasets. The maximum normalized discounted cumulative gain at 10 (NDCG@10), precision at 10 (p@10) partial and p@10 strict of 0.89, 0.78 and 0.61, respectively, were obtained using the proposed method after manual evaluation by five researchers. As per the best of our knowledge, this is the first study of its kind on content-based dataset recommendation. We hope that this system will further promote data sharing, offset the researchers’ workload in identifying the right dataset and increase the reusability of biomedical datasets.

**Database URL**: http://genestudy.org/recommends/#/

## Introduction

In the Big Data era, extensive amounts of data have been generated for scientific discoveries. However, storing, accessing, analyzing and sharing a vast amount of data are becoming major bottlenecks for scientific research. Furthermore, making a large number of public scientific data findable, accessible, interoperable and reusable is a challenging task.

The research community has devoted substantial effort to enable data sharing. Promoting existing datasets for reuse is a major initiative that gained momentum in the past decade (1). Many repositories and knowledge bases have been established for specific types of data and domains. Gene Expression Omnibus (GEO) (https://www.ncbi.nlm.nih.gov/geo/), UKBioBank (https://www.ukbiobank.ac.uk/), ImmPort (https://www.immport.org/shared/home) and TCGA (https://portal.gdc.cancer.gov/) are some examples of repositories for biomedical datasets. DATA.GOV archives the U.S. Government’s open data related to agriculture, climate, education, etc. for research use. However, a researcher looking for previous datasets on a topic still has to painstakingly visit all the individual repositories to find relevant datasets. This is a tedious and time-consuming process.

An initiative was taken by the developers of DataMed (https://datamed.org) to solve the aforementioned issues for the biomedical community by combining biomedical repositories together and enhancing the query searching based on advanced natural language processing (NLP) techniques (1, 2). DataMed indexes provides the functionality to search diverse categories of biomedical datasets (1). The research focus of this last work was retrieving datasets using a focused query. In addition to that biomedical and healthCAre Data Discovery Index Ecosystem (bioCADDIE) dataset retrieval challenge was organized in 2016 to evaluate the effectiveness of information retrieval (IR) techniques in identifying relevant biomedical datasets in DataMed (3). Among the teams participated in this shared task, use of probabilistic or machine learning based IR (4), medical subject headings (MeSH) term based query expansion (5), word embeddings and identifying named entity (6), and re-ranking (7) for searching datasets using a query were the prevalent approaches. Similarly, a specialized search engine named Omicseq was developed for retrieving omics data (8).

Google Dataset Search (https://toolbox.google.com/datasetsearch) provides the facility to search datasets on the web, similar to DataMed. While DataMed indexes only biomedical domain data, indexing in Google Dataset Search covers data across several domains. Datasets are created and added to repositories frequently, which makes it difficult for a researcher to know and keep track of all datasets. Further, search engines such as DataMed or Google Dataset Search are helpful when the user knows what type of dataset to search for, but determining the user intent in web searches is a difficult problem due to the sparse data available concerning the searcher (9). To overcome the aforementioned problems and make dataset search more user-friendly, a dataset recommendation system based on a researcher’s profile is proposed here. The publications of researchers indicate their academic interest, and this information can be used to recommend datasets. Recommending a dataset to an appropriate researcher is a new field of research. There are many datasets available that may be useful to certain researchers for further exploration, and this important aspect of dataset recommendation has not been explored earlier.

Recommendation systems, or recommenders, are an information filtering system that deploys data mining and analytics of users’ behaviors, including preferences and activities, for predictions of users’ interests on information, products or services. Research publications in recommendation systems can be broadly grouped as content-based or collaborative filtering recommendation systems (10). This article describes the development of a recommendation system for scholarly use. In general, developing a scholarly recommendation system is both challenging and unique because semantic information plays an important role in this context, as inputs such as title, abstract and keywords need to be considered (11). The usefulness of similar research article recommendation systems has been established by the acceptance of applications such as Google Scholar (https://scholar.google.com/), Academia.edu (https://www.academia.edu/), ResearchGate (https://www.researchgate.net/), Semantic Scholar (https://www.semanticscholar.org/) and PubMed (https://www.ncbi.nlm.nih.gov/pubmed/) by the research community.

Dataset recommendation is a challenging task due to the following reasons. First, while standardized formats for dataset metadata exist (12), no such standard has achieved universal adoption, and researchers use their own convention to describe their datasets. Further, many datasets do not have proper metadata, which makes the prepared dataset difficult to reuse/recommend. Second, there are many dataset repositories with the same dataset in different formats, making recommendation a challenging task. Additionally, the dataset recommendation system should be scalable to the increasing number of online datasets. We cast the problem of recommending datasets to researchers as a ranking problem of datasets matched against the researcher’s individual publication(s). The recommendation system can be viewed as an IR system where the most similar datasets can be retrieved for a researcher using his/her publications.

Data linking or identifying/clustering similar datasets have received relatively less attention in research on recommendation systems. Previous work on this topic includes (13–15). Reference (13) defined dataset recommendation as to the problem of computing a rank score for each of a set of target datasets (*D*_*T*_) so that the rank score indicates the relatedness of *D*_*T*_ to a given source dataset (*D*_*S*_). The rank scores provide information on the likelihood of a *D*_*T*_ to contain linking candidates for *D*_*S*_. Reference (15) proposed a dataset recommendation system by first creating similarity-based dataset networks, and then recommending connected datasets to users for each dataset searched. Despite the promising result this approach suffers from the cold start problem. Here cold start problem refers to the user’s initial dataset selection, where the user has no idea what dataset to select/search. If a user chooses a wrong dataset initially, then the system will always recommend wrong datasets to the user.

Some experiments were performed to identify datasets shared in the biomedical literature (16–18). Reference (17) identified data shared in biomedical literature articles using regular expression patterns and machine learning algorithms. Reference (16) identified datasets in social sciences papers using a semi-automatic method. The last system reportedly performed well (F-measure of 0.83) in finding datasets in the **da|ra** dataset registry. Different deep learning methods were used to extract the dataset mentions in publication and detect mention text fragment to a particular dataset in the knowledge base (18). Further, a content-based recommendation system was developed for recommending literature for datasets in (11), which was the first step toward developing a literature recommendation tool by recommending relevant literature for datasets.

This article proposes a dataset recommender that recommends datasets to researchers based on their publications. We collected dataset metadata (title and summary) from GEO and researcher’s publications (title, abstract and year of publication) from PubMed using name and curriculum vitae (CV) for developing a dataset recommendation system. A vector space model (VSM) is used to compare publications and datasets. We propose two novel ideas:

- A method for representing researchers with multiple vectors reflecting each researcher’s diverse interests.
- A system for recommending datasets to researchers based on their research vectors.

For the datasets, we focus on GEO (https://www.ncbi.nlm.nih.gov/geo/). GEO is a public repository for high-throughput microarray and next-generation sequence functional genomics data. It was found that an average of 21 datasets was added daily in the last 6 years (i.e. 2014–19). This gives a glimpse of the increasing number of datasets being made available online, considering that there are many other online data repositories as well. Many of these datasets were collected at significant expense, and most of these datasets were used only once. We believe that reusability of these datasets can be improved by recommending these to appropriate researchers.

Efforts on restructuring GEO have been performed by curating available metadata. In reference (19), the authors identified the important keywords present in the datasets descriptions and searched other similar datasets. Another task on restructuring the GEO database, ReGEO (http://regeo.org/) was developed by (20), who identified important metadata such as time points and cell lines for datasets using automated NLP techniques.

We developed this dataset recommendation system for researchers as a part of the dataset reusability platform (GETc Research Platform(http://genestudy.org/)) for GEO developed at the University Texas Health Science Center at Houston. This website recommends datasets to users using their publications.

The rest of the article is organized in the following manner. Section 2 provides an overview of GEO datasets and researcher publications. Methods used for developing the recommendation system and evaluation techniques used in this experiment are described in Section 3. Section 4 describes results. Section 5 provides a discussion. Finally, conclusion and future directions are discussed in Section 6.

## Data

The proposed dataset recommendation system requires both dataset metadata and the user profile for which datasets will be recommended. We collected metadata of datasets from the GEO repository, and researcher publications from PubMed using their names and CVs. The data collection methods and summaries of data are discussed next.

### GEO Datasets

**Figure 1.**
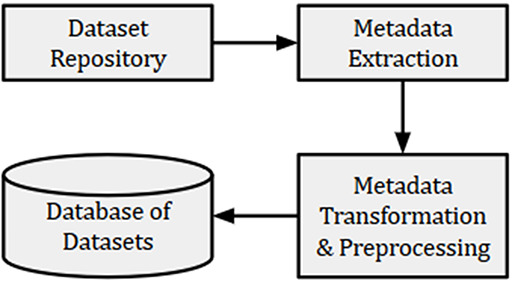
Histogram of datasets submitted to GEO based on datasets collected on December 18, 2019

GEO is one of the most popular public repositories for functional genomics data. As of December 18, 2019, there were 122 222 series of datasets available in GEO. Histograms of datasets submitted to GEO per day and per year as presented in Figure 1 showed an increasing trend of submitting datasets to GEO, which justified our selection of this repository for developing the recommendation system.

**Figure 2.**
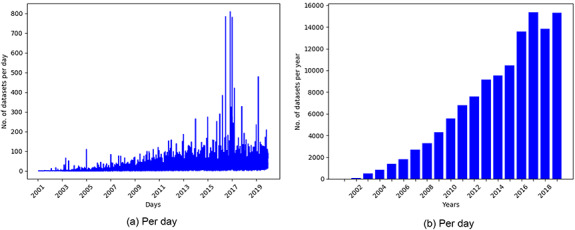
Overview of dataset indexing pipeline

**Table 1.** Statistics of datasets collected from GEO

**Datasets**	122 222
**Datasets with articles**	89 533
**Total articles**	92 884 (Mean : 0.76, Max : 10, Unique : 61 228)

For the present experiment, metadata such as title, summary, submission date and name of dataset creator(s) were collected from GEO and indexed in a database, as shown in Figure 2. We also collected the PMIDs of articles associated with each dataset. However, many datasets did not have articles associated with them. The detailed information of collected datasets is presented in Table 1. Out of a total of 122 222 GEO datasets, 89 533 had 92 884 associated articles, out of which 61 228 were unique. The maximum number of articles associated with the datasets (‘GSE15907’ and ‘GSE31312’) was 10. These articles were used to remove the publications that were not related to GEO. Further, we used the GEO-related publications for building word embeddings to be used for subsequent text normalization as outlined in Section 3.

### Researcher publications

A researcher’s academic interest can be extracted from publications, grants, talks, seminars and much more. All this information is typically available in the CV, but it is presented in the form of titles/short texts. Here, short texts imply limited information. Further, lack of standardization in CV formats poses challenges to parse the CVs. In this work, an alternative approach was undertaken, which is outlined next.

Title and year of the researcher’s publications were present in the CV. However, we required title, abstract and year of publication for our experiment. A researcher’s list of publications (titles and abstracts) are easier to get from web sources such as Google Scholar, PubMed, Semantic Scholar and others. Unfortunately, the full texts of most scientific articles are not publicly available. Thus, for the present experiment, we used only the title and abstract of publications in identifying the researcher’s areas of research.

**Figure 3.**
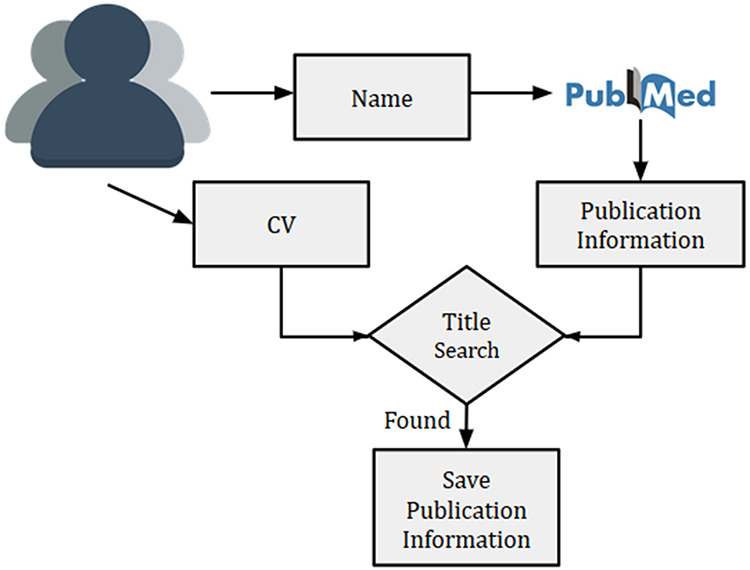
Overview of researcher’s publication extraction system to remove the author disambiguation

Given a researcher, we searched the researcher’s name in PubMed using Entrez API (https://www.ncbi.nlm.nih.gov/books/NBK25 501/) and collected all the publications. Multiple researchers with exact same name might exist, thus, querying the name in PubMed might sometime result in publications from other researchers as well. This is a typical challenge of author disambiguation. However, there are a few attempts that have been undertaken to resolve the issue of author disambiguation, and one of them is ORCID (https://orcid.org). A researcher needs to provide ORCID id to access his/her ORCID details. However, to the best of our knowledge, many researchers in the biomedical domain did not have an associated ORCID account. Thus we used a simple method to disambiguate the authors by using their CVs. Initially, the recommendation system prompts a researcher to provide his/her name and a CV (or list of publications). Next, we collected the publications (titles, names, MeSH terms and year of publication) for a researcher from PubMed by searching his/her name. For removing the publications of other authors with the same name, titles of all collected publications from PubMed were matched against the titles present in the CV. In the case of a match, publications were kept for further processing. An overview of the technique used for the researcher’s publication collection is provided in Figure 3.

One of the limitations of the above publication collection method is that the publications could not be collected if they were not listed in PubMed. Further, the datasets used in the present experiments were from the biomedical domain, and the publications not listed in PubMed were less pertinent to biomedical datasets. For example, someone’s biomedical interests (in PubMed) may be more reliable markers for biomedical datasets than a theoretical computer science or statistics paper. Another downside is if the researcher’s CV may not be fully up-to-date.

## Methods and evaluation

This section describes how the two main objects of interest (datasets and publications of researchers) were embedded in a vector space and then how these vectors were compared in order to make recommendations. First, both datasets and papers were treated as text objects: the text of a dataset includes its title and summary, while the text of a paper includes its title and abstract. Pre-processing was performed on both a researcher’s publications and datasets by removing the low-value stopwords, links, punctuation and junk words. Further, the nltk WordNet lemmatizer (https://www.nltk.org/_modules/nltk/stem/wordnet.html) was used to get the root forms of the words. Next, we describe the methods used for converting datasets and researchers into vectors.

### Dataset vector generation

VSMs can be built from text in a variety of ways, each of which has its distinct advantages and thus merit experimentation. For the present experiment, we used TF-IDF because it achieved better results for related literature recommendation for datasets in (11).

**TF-IDF**: For vocabulary *W*, each unique word *w* ∈ *W* is assigned a score proportional to its frequency in the text (term frequency, TF) and its inverse frequency in the full collection (inverse document frequency, IDF). We tuned parameters such as minimum document frequency (min-df) and maximum n-gram size. For the present study, we kept maximum n-gram size = 2 (i.e. unigrams and bigrams) as including the higher n-gram increases the sparsity as well as computational complexity.

We converted each dataset into a vector using TF-IDF. For each dataset, the title and summary were preprocessed and normalized and then converted into a single vector. Finally, each publication vector (or publication cluster vector) is compared with dataset vectors to generate the recommendation score. Different methods for representing a researcher’s papers as vectors are discussed next.

### Researcher vector generation

#### Baseline method

For the baseline method, we combined multiple text-derived paper vectors into a single researcher vector (*v*_*r*_) in the same vector space using Equation (1):

(1) \begin{equation*} v_r = \frac{1}{N_r} \sum_{p\in P_r} \lambda_p v_p \end{equation*}

where *P*_*r*_ is the set of papers of a researcher *r*; *N*_*r*_ is the total number of papers of that researcher, and it acts as a normalization term; *v*_*p*_ is the vector for a single paper *p* using TF-IDF; *λ*_*p*_ is a recency penalty to favor more recent papers (thus better reflecting the researcher’s current interest).

It is evident that a researcher will be interested in datasets recommended for his/her current work rather than the work performed a few years back. Thus, we penalized each of the paper vectors from a different year, as stated in Equation (2):

(2) \begin{equation*} \lambda_p = \frac{1}{e^{kt}} \end{equation*}

where *t* is the difference between the current year and year of publication. *k* is the decaying function to decrease the rate proportional to its current value, and for the present study, we kept *k*=0.05.

#### . Multi-interest dataset recommendation (MIDR)

The baseline method for creating a researcher vector may be helpful for new researchers without many publications, whereas an established researcher may have multiple areas of expertise with multiple papers in each. Also, if the number of papers is imbalanced in multiple areas, then the above baseline method may not work. With a highly imbalanced set of publications this would obviously bias dataset recommendation to the dominant interest. For a more balanced set of interests that are highly dispersed, this mixture would result in the ‘centroid’ of these interests, which could be quite distinct from the individual interests. Both these cases are undesirable. The centroid of a researcher’s interests may not be of much interest to them (e.g. a researcher interested in *mouse genomics* and *HIV vaccines* may not be interested in *mouse vaccines*).

For example, initial experiments were performed on Researcher 1 (mentioned later in Section 4), and it was observed that the datasets recommended for a researcher were biased toward a single research area with the largest number of publications. For example, Researcher 1 has a dominant number of publications on *HIV* and the baseline system recommends only *HIV* datasets, even if Researcher 1 has multiple research areas.

A critical limitation of the above baseline approach is that researchers can have multiple areas of expertise. We can easily build multiple vectors, each corresponding to a different expertise if we know how to properly group/cluster a researcher’s papers according to expertise or topic. However, parametric methods such as k-means clustering and latent Dirichlet allocation require specifying a priori how many clusters/topics to utilize. Generalizing the number of clusters is not possible due to a varying number of publications of researchers. Instead, our insight is that the more publications a researcher has, the more interests or areas of expertise he/she likely has as well, but this should be modeled as a ‘soft’ constraint rather than a ‘hard’ constraint. We propose to employ the non-parametric Dirichlet Process Mixture Model (DPMM) (21) to cluster papers into several groups of expertise.

**Figure 4.**
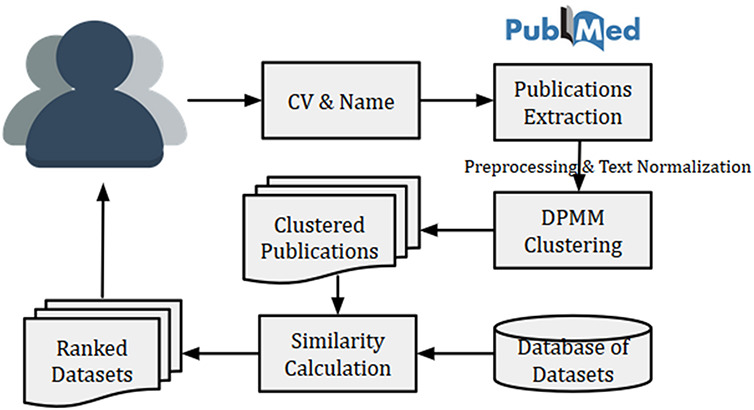
High level architecture of proposed dataset recommendation

**DPMM**: We employed a Gibbs Sampling-based Dirichlet Process Mixture Modeling for text clustering. DPMM offers the following advantages over its traditional counterparts. First, the number of text clusters need not be specified; second, it is relatively scalable and third, it is robust to outliers (22). The technique employs a collapsed Gibbs Sampling mechanism for Dirichlet process models wherein the clusters are added or removed based on the probability of a cluster associating with a given document. The scalability of the technique stems from the fact that word frequencies are used for text clustering. This reduces the computational burden significantly, considering the large number of samples associated with text processing problems. Further, the optimal number of clusters is likely to be chosen, as clusters with low association probability with documents are eliminated, and new clusters are created for documents that do not belong to selected clusters with high probability. For example, if a cluster *c*_1_ contains five documents, each with low association probability, then the cluster *c*_1_ is eliminated, and new clusters are initialized. In DPMM, the decision to create a new cluster is based on the number of papers to be clustered and the similarity of a given paper to previously clustered papers. Thus, researchers with many papers but few interests can still result in fewer clusters than a researcher with fewer papers but more interests. For example, our evaluation includes two researchers, one with 53 papers and one with 32; however, the DPMM resulted in five and six clusters, respectively. After clustering, we created a pseudo-researcher for each cluster using Equation (1), though one that can be tied back to the original researcher. The recommendation system uses these pseudo-researchers in its similarity calculations along the same lines as described above. Further, the *α* parameter was tuned to control the number of clusters (22). We describe tuning of the *α* parameter in Section 3.4.

**Text normalization**: Text normalization plays an important role in improving the performance of any NLP system. We also implemented text normalization techniques to improve the efficiency of the proposed clustering algorithm. We normalized similar words by grouping them together and replacing them with the most frequent words in the same word group. For example, *HIV, HIV-1, HIV/AIDS* and *AIDS* were replaced with the most frequent word *HIV*. For identifying similar words, we trained a word2vec model on the articles from PubMed using Gensim (https://radimrehurek.com/gensim/). The datasets are related to gene expressions, while the articles collected from PubMed contain a variety of topics related to biomedicine and life sciences which may not be suitable for building a word embedding in the current study (since some of these articles are highly unrelated to the type of information in GEO). The articles before 1998 were removed as the research on micro-array data started during that year (23). The publications related to GEO are filtered using the MeSH terms. We also developed a MeSH term classification system for those publications without MeSH terms. More details on GEO related publications filtering can be found in (11).

The similar words were identified using the most_similar function of word2vec. We only considered the top five similar words for each word using most_similar function. The normalized text was used for clustering. It was observed from the initial experiments that the text normalization improved clustering and resulted in the reduced number of clusters using DPMM.

### Dataset recommendation

The most similar datasets can be recommended to researchers simply by comparing the cosine similarity of the researcher and dataset vectors using Equation (3):

(3) \begin{equation*} {\rm sim}(r,d) = \cos(v_r, v_d) = \frac{v_r \cdot v_d}{||v_r|| \: ||v_d||} \end{equation*}

where *D* is all the datasets that can be recommended to researcher *r*; $\cos(v_r, v_d)$ is the cosine similarity between researcher vector (*v*_*r*_) and dataset vector (*v*_*d*_).

The high-level system architecture of the dataset recommendation system is shown in Figure 4. This dataset recommendation system is initiated by a researcher (user) by submitting his/her name and CV (or list of publications). The name is searched in PubMed for publication details, and then titles of publications from PubMed were matched with publication titles in CV. The matched publications are then clustered using DPMM to identify research fields of the researcher. Finally, the top similar datasets are recommended using the calculated cosine similarity between the researcher vector (or researcher’s cluster vector) and dataset vectors. The researcher vector (or researcher’s cluster vector) is calculated using Equation (1).

Three dataset recommendation systems are evaluated in this article: a baseline method using the researcher’s vector generation method and two proposed methods using the proposed researcher’s vector generation method.

#### Baseline system

The baseline system uses the researcher’s vector using Equation (1) of the baseline method in Section 3.2.1. The top datasets are recommended after calculating the cosine similarity between the researcher’s vector and dataset vectors. This system reflects only one research field for each researcher.

#### MIDR System

The cluster vectors are generated using the modified Equation (1). Here, cluster-specific research area vectors are created for each researcher, instead of a single vector for each researcher as in baseline system. Papers in a single cluster are multiplied with their recency factors and summed. Then, the summation was divided the number of papers in that cluster.

This system uses multiple pseudo vectors for multiple clusters of a researcher ($v_{c_i}$ for *i*^th^ cluster), indicating different research fields that a researcher might have, as mentioned in Section 3.2.2.

This system compares each cluster vector with the dataset vectors and recommends the top datasets by computing the cosine similarity among them. Finally, it merges all the recommended datasets in a round-robin fashion for all the clusters, so that the researcher is able to see various datasets related to different research fields together.

#### MIDR System (Separate)

This system is an extension of our proposed MIDR system. Some researchers liked the way recommended datasets were merged. However, other researchers wanted dataset recommendations for each cluster separately. For this reason, another system was developed where the recommended datasets were shown separately for each research cluster, allowing researchers to obtain different recommended datasets for different research interests.

**Table 2.** Number of clusters with varying *α* values for proposed *α* based on our initial evaluation. Abbreviations: P: Proposed, a: total number of clusters, b: number of clusters which contains more than one paper, c: number of clusters which contains only one paper

Researcher ID (No. of papers)	Number of clusters (a, b, c) for different *α* values					
	*α* = 0.3	*α* = 1.0	*α* = 2.0	*α* = 3.0	*α* = 10.0	*α* = P
**1 (53)**	16, 9, 7	8, 7, 1	6, 3, 3	5, 2, 3	3, 2, 1	8, 5, 3 (1.37)
**2 (32)**	15, 10, 5	10, 8, 2	8, 6, 2	7, 5, 2	4, 2, 2	8, 6, 2 (1.77)
**3 (48)**	15, 8, 7	9, 4, 5	3, 3, 0	6, 2, 4	2, 1, 1	6, 3, 3 (1.44)
**4 (22)**	15, 4, 11	10, 5, 5	6, 4, 2	5, 3, 2	4, 1, 3	7, 4, 3 (2.13)
**5 (7)**	5, 2, 3	5, 2, 3	4, 3, 1	5, 2, 3	3, 3, 0	5, 2, 3 (3.8)
**6 (76)**	11, 10, 1	6, 5, 1	3, 3, 0	2, 2, 0	2, 1, 1	5, 5, 0 (1.14)
**7 (13)**	7, 3, 4	5, 4, 1	3, 3, 0	2, 2, 0	2, 2, 0	2, 2, 0 (2.77)
**8 (193)**	16, 15, 1	6, 5, 1	8, 4, 4	5, 2, 3	4, 1, 3	7, 6, 1 (0.72)
**9 (291)**	23, 23, 0	9, 8, 1	6, 6, 0	6, 3, 3	3, 1, 2	14, 14, 0 (0.59)
**10 (54)**	20, 11, 9	12, 10, 2	9, 8, 1	6, 6, 0	2, 2, 0	9, 8, 1 (1.36)

### Tuning the *α* parameter

A researcher with a higher number of publications is more likely to have more research interests. In this paper, research interests are represented as clusters, expressed as vectors. A Dirichlet process is non-parametric because, in theory, there can be an infinite number of clusters. By changing the *α* parameter, DPMM can vary the number of clusters. The *α* value is inversely related to the number of clusters, i.e. decreasing the *α* parameter in DPMM may increase the number of output clusters. Therefore, we propose an *α* value, which is also inversely related to the number of research publications. Further, the *α* value must stabilize after a certain threshold to avoid the formation of too many clusters, and it must be generalized to the number of publications. To this end, *α* is calculated as follows:

(4) \begin{equation*} \alpha = \frac{10}{\sqrt{N}} \end{equation*}

where *N* is the total number of papers for a researcher. The *α* value is proposed based on manually observing the clusters and collecting feedback from different researchers. Apart from inherent requirements for setting *α*, Equation (4) maintains a reasonable number of clusters, which was found useful by most of the evaluators.

Different *α* values and their corresponding number of clusters are provided in Table 2. The number of clusters are divided into three categories: (a) total number of clusters, (b) number of clusters which contains more than one paper, (c) number of clusters which contains only one paper. We removed the clusters with one paper and used the clusters with two or more papers for recommending datasets. We observed that the number of clusters did not entirely depend upon the number of papers, a researcher had. Moreover, it largely reflected the number of research fields that the researcher participated in. For example, Researcher 2 had fewer publications than Researcher 1 and Researcher 3, but the number of clusters was more than the others. This shows that non-parametric clustering is a good technique for segmenting research areas.

### Evaluation

#### Clustering

There is no existing labeled clustered publication datasets available for automatic evaluation. Again, manually evaluating the clusters was a time and resource-consuming task. It might be biased as the evaluation depends upon different judgments for different researchers. Thus, we implemented K-Means for comparing to the proposed DPMM. The automatic cluster comparison was performed using inter- and intra-cluster cosine similarity (IACCS) of words and MeSH terms in the publications, separately. IACCS was the mean cosine similarity of words or MeSH terms for each pair of papers in a given cluster. Considering a cluster of size *n* ($X=\{x_1, x_2, \dots x_n\}$), the IACCS can be formulated using Equation (5):

(5) \begin{equation*} {\rm IACCS} = \frac{1}{C^{\,n}_2}\sum_{i=1}^{n-1}\sum_{j=i+1}^{n}\cos(x_i, x_j) \end{equation*}

where, *x*_*i*_ and *x*_*j*_ are the list of MeSH terms or words of the *i*^th^ and *j*^th^ paper, respectively, and $\cos(x_i, x_j)$ is the cosine similarity between them. Finally, the mean of IACCS was calculated using the IACCS of individual clusters.

We computed the mean cosine similarity between words or MeSH terms of papers within clusters to calculate the inter-cluster cosine similarity (ICCS). Considering *n* clusters ($c_1, c_2, \dots c_n$), ICSS can be formulated using Equation (6):

(6) \begin{equation*} {\rm ICCS} = \frac{1}{C^{\,n}_2}\sum_{i=1}^{n-1}\sum_{j=i+1}^{n}\cos(c_i, c_j) \end{equation*}

where, *c*_*i*_ and *c*_*j*_ are the list of MeSH terms or words of all the papers in the *i*^th^ and *j*^th^ clusters, respectively, and $\cos(c_i, c_j)$ is the cosine similarity between them.

For the baseline comparison, publication vectors are created using TF-IDF, then K-Means is used to compute the publication clusters. K-Means is a parametric unsupervised clustering. We implemented K-Means with two and five clusters separately for comparison purposes. On the other hand, the tuning parameter proposed for DPMM resulted in a variable number of clusters for different researchers, and these clusters were used for comparison.

#### Recommendation system

**Table 3.** Mean IACCS and ICCS for K-Means and DPMM (with different cluster sizes as mentioned in Table 2).

	K-Means					
	Clusters = 2		Clusters = 5		DPMM	
	Words	MeSH terms	Words	MeSH terms	Words	MeSH terms
Researcher ID (No. of papers)	IACSS, ICSS	IACSS, ICSS	IACSS, ICSS	IACSS, ICSS	IACSS, ICSS	IACSS, ICSS
**1 (53)**	0.14, 0.37	0.12, 0.56	0.20, 0.26	0.12, 0.27	0.22, 0.19	0.20, 0.32
**2 (32)**	0.09, 0.37	0.09, 0.42	0.14, 0.16	0.14, 0.23	0.22, 0.11	0.11, 0.12
**3 (48)**	0.16, 0.43	0.16, 0.56	0.16, 0.25	0.16, 0.29	0.24, 0.22	0.17, 0.37
**4 (22)**	0.12, 0.24	0.09, 0.51	0.21, 0.11	0.15, 0.20	0.20, 0.10	0.16, 0.15
**5 (7)**	0.16, 0.13	0.14, 0.19	0.18, 0.04	0.11, 0.08	0.45, 0.08	0.28, 0.13
**6 (76)**	0.17, 0.54	0.19, 0.64	0.20, 0.31	0.22, 0.36	0.20, 0.18	0.23, 0.19
**7 (13)**	0.33, 0.14	0.47, 0.30	0.32, 0.17	0.45, 0.30	0.34, 0.14	0.47, 0.30
**8 (193)**	0.10, 0.61	0.16, 0.76	0.17, 0.31	0.21, 0.60	0.17, 0.25	0.21, 0.43
**9 (291)**	0.07, 0.55	0.09, 0.64	0.09, 0.40	0.11, 0.54	0.23, 0.13	0.14, 0.19
**10 (54)**	0.07, 0.31	0.15, 0.57	0.11, 0.19	0.23, 0.33	0.17, 0.10	0.20, 0.24

Being a novel task, no prior ground truth annotations exist for publication-driven dataset recommendation. Thus, we performed a manual evaluation for each developed dataset recommendation system. We asked researchers to rate each retrieved dataset based on their publications or publication clusters. The researchers included in this study have already worked on the datasets from GEO and published papers on these datasets. The rating criterion was how likely they want to work on the retrieved datasets. We asked them to rate using one to three ‘stars’, with three stars being the highest score. Later, normalized discounted cumulative gain (NDCG) at 10 and Precision at 10 (P@10) were calculated to evaluate different systems. The ratings are:

- **1 star [not relevant]**: This dataset is not useful at all.
- **2 star [partially relevant]**: This dataset is partially relevant to the publication cluster. The researcher has already used this dataset or maybe work on it in the future.
- **3 star [most relevant]**: This dataset is most relevant to the publication cluster, and the researcher wants to work on this dataset as soon as possible.

The primary evaluation metric used in this work is NDCG, which is a family of ranking measures widely used in IR applications. It has advantages compared to many other measures. First, NDCG allows each retrieved document to have a graded relevance, while most traditional ranking measures only allow binary relevance (i.e. each document is viewed as either relevant or not relevant). This enables the three-point scale to be directly incorporated into the evaluation metric. Second, NDCG involves a discount function over the rank while many other measures uniformly weight all positions. This feature is particularly important for search engines as users care about top-ranked documents much more than others (24). NDCG is calculated as follows:

(7) \begin{equation*} {\rm Normalized}\ DCG_p = \frac{\sum_{i=1}^{p}{\frac{rating(i)}{\log_2(i + 1)}}}{\sum_{i=1}^{|REL|}{\frac{rating(i)}{\log_2(i + 1)}}} \end{equation*}

where *rating*(*i*) is the *i*th dataset rating provided by users. For the present study, we set *p* = 10 for the simplicity of manual annotation.

The NDCG@10 for the baseline and MIDR systems is calculated using the ratings of only the top ten retrieved datasets. For the MIDR system (separate), there were multiple publication clusters for a single user, and for each publication cluster we recommended datasets separately. NDCG@10 was calculated for each publication cluster using the top ten datasets and later averaged to get a final NDCG@10 for a single researcher. For NDCG@10 calculation, the 1-star, 2-star and 3-star are converted to 0, 1 and 2, respectively. We also calculated P@10 (strict and partial) for the baseline and proposed systems. Strict considers only 3-star, while partial considers both 2- and 3-star results. The results presented in this study were evaluated using a total of five researchers (with an average of 32 publications) who already worked on GEO datasets. This is admittedly a small sample size, but is large enough to draw coarse comparisons on this novel task.

## Results

We compared DPMM clustering with K-Means as mentioned in Section 3.5. ICCS and mean IACCS values for different clustering methods are presented in Table 3. In general, higher mean IACSS and lower ICCS generally indicate better clustering. However, this is not always the case, especially when the number of clusters are small, and each cluster contains multiple publications for a single researcher. In this situation, the IACSS for individual cluster decreases after being divided by the number of publication pairs in each cluster. Furthermore, DPMM and K-Means were comparable when the number of clusters produced by both were close to each other. For all the cases, DPMM had higher mean IACSS and lower ICCS than K-Means using words. This suggests that DPMM was well-suited for clustering a researcher’s publications into multiple research fields.

Researcher-specific results of the dataset recommendation system are shown in Table 4. The results for individual researchers are listed for all the systems. Metric-specific average results for all the systems are also shown in Table 4. The baseline system did not have any publication clusters and all publications were vectorized using Equation (1). Next, the top ten similar datasets were used to evaluate the results of the baseline system and it obtained the average NDGC@10, P@10 (P) and P@10 (S) of 0.80, 0.69 and 0.45, respectively.

The proposed MIDR system obtained the average NDCG@10, P@10 (P) and P@10 (S) of 0.89, 0.78 and 0.61, respectively. The proposed MIDR (separate) system obtained the average NDGC@10, P@10 (P) and P@10 (S) of 0.62, 0.45 and 0.31, respectively. For calculating NDCG@10 and P@10 in the proposed MIDR (separate) system, individual cluster scores were calculated first, and then divided by the total number of clusters.

**Table 4.** NDCG@10, partial and strict P@10 values of the different dataset recommendation systems based on three evaluators. Abbreviations: Partial: P; Strict: S

Researcher ID (No. of papers)	Baseline			MIDR			MIDR (separate)		
	NDCG@10	P@10 (P)	P@10 (S)	NDCG@10	P@10 (P)	P@10 (S)	NDCG@10	P@10 (P)	P@10 (S)
**1 (53)**	0.82	0.80	0.30	0.92	0.90	0.40	0.74	0.56	0.32
**2 (32)**	0.81	0.76	0.50	0.95	0.80	0.70	0.52	0.38	0.20
**3 (48)**	0.76	0.60	0.28	0.80	0.60	0.34	0.60	0.25	0.13
**4 (22)**	0.78	0.48	0.36	0.80	0.60	0.60	0.48	0.34	0.22
**5 (7)**	0.85	0.80	0.80	1.00	1.00	1.00	0.78	0.70	0.70
**Average**	**0.80**	**0.69**	**0.45**	**0.89**	**0.78**	**0.61**	**0.62**	**0.45**	**0.31**

## Discussion

The proposed MIDR system performed better than the baseline system. The MIDR system recommended a variety of datasets involving multiple clusters/research fields as opposed to the baseline system recommended datasets from a single research field with the maximum number of publications.

Performances of the baseline and proposed MIDR (separate) systems could not be directly compared. Evaluation of the MIDR (separate) system was performed over multiple clusters with ten datasets recommended for each cluster. In contrast, evaluation of the baseline system was performed only on 10 datasets, for example, for Researcher 1 in Table 4, evaluations of baseline system and MIDR (separate) system were performed on 10 and 50 datasets, respectively. There were other advantages of the MIDR (separate) system over the baseline system, irrespective of higher NDCG@10 for the latter. The baseline system had a bias toward a specific research field which was eliminated in the MIDR (separate) system. For Researchers 1 and 2 in Table 4, the datasets recommended by the baseline system were found in the results of two clusters/research fields (which had the maximum number of publications) in the proposed MIDR (separate) system. However, for Researcher 3 in Table 4, recommended datasets of the baseline system were found in the results of only one research field (with the maximum number of publications) in the proposed MIDR (separate) system.

For Researcher 1 in Table 4, there were 31 papers with *HIV* keywords and those papers were not published recently. We penalized the papers according to the year of publication for all methods. However, the top datasets contained *‘HIV’* or related keywords for the baseline method. We manually checked the top 100 results and found that those were relevant to *HIV*. Whereas, the proposed MIDR system clustered the publications into different groups (such as *HIV, Flu/Influenza*, and others), which resulted in recommendations for different research fields. Therefore, Researcher 1 had the flexibility to choose the datasets after looking at the preferred clusters in the proposed MIDR or MIDR (separate) system.

Similarly, the results of the MIDR and MIDR (separate) systems could not be directly compared. Evaluation of the MIDR system was performed based on 10 datasets recommended for each researcher, whereas evaluation of MIDR (separate) system was performed based on 10 recommended datasets for each research field (cluster), which could be more than 10 datasets if a researcher had more than one research fields (clusters). Hence, the NDCG@10 and P@10 scores of MIDR (separate) system were less than the MIDR system.

For researchers looking to find specific types of datasets, a keyword-based IR system might be more useful. For researcher who generally wanted to find datasets related to their interests, but did not have a particular interest in mind, could benefit from our system. For instance, if a researcher wanted a regular update of datasets relevant to their interest, our method would be better suited. However, this proposed system may not be useful to early-stage researchers due to fewer publications. They may take advantage of the available dataset retrieval systems such as DataMed, Omicseq and Google Dataset Search; or the text-based dataset searching that we provided on the website.

### Error analysis

For some clusters, evaluators rated all recommended datasets as one star. In most of these cases, we observed that the research field of that cluster was out of the scope of GEO. In this case, the NDCG@10 score was close to 1, but the P@10 score was 0. This may be one of the reasons why NDCG@10 scores were much higher compared to P@10 scores.

**Figure 5.**
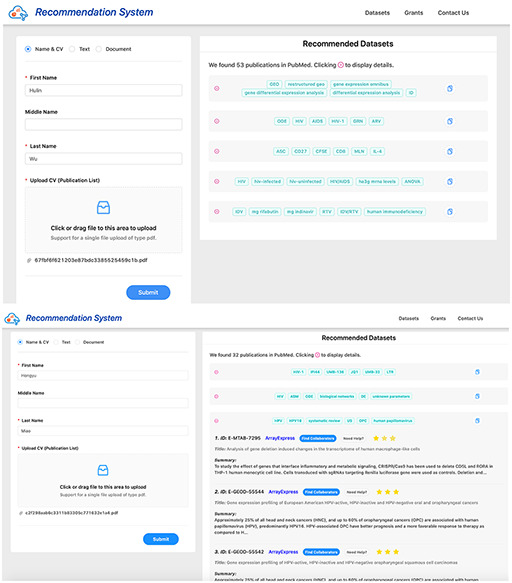
Screenshots of dataset recommendation system Researcher 1 (up) and Researcher 2 (down).

Initially, we had not identified whether the clusters were related to GEO or not. We recommended datasets for these unrelated clusters. For example, Researcher 2 in Table 4 had a paper cluster which was related to statistical image analysis. For this specific cluster, Researcher 2 rated all the recommended datasets as one star, which reduced the scores of the systems.

Later, we identified a threshold by averaging the similarity scores of publications and datasets for each cluster, and were able to remove the clusters which were not related to GEO. The threshold was set to 0.05 for the present study, i.e. a cluster was not considered for evaluation or showing recommendation if the average similarity score of the top 10 datasets for that cluster was less than or equals to 0.05. This threshold technique improved the results of proposed systems by 3% for Researcher 2. However, a thorough investigation on threshold involving datasets from different biomedical domains is needed for future work.

Further, a dislike button for each cluster may be provided, and users may press the dislike button if that cluster is not related to GEO datasets. Later, this information can be used to build a machine learning-based system to identify and remove such clusters from further processing. This will improve the usefulness and reduce time complexity of the proposed recommendation system.

### . Limitations

The researchers’ names are searched in PubMed to collect their publications. Many recent conference/journal publications are not updated in PubMed. Further, if the researcher has most of his/her publications that did not belong to the biomedical domain, then there is a low chance of getting those papers in PubMed. This makes the dataset recommendation task harder. Authors might later be able to include a subset of their non-PubMed articles for consideration in dataset recommendation (e.g. bioRxiv preprints), but this work is currently limited to PubMed publications only.

We used PubMed name search to find the titles of a researcher’s papers. Finally, the titles were matched with the text in the CV to get publications. If there is any typo in the CV, then that publication would be rejected from being processed in further steps. As we do not fully parse the CV, instead just performing string matching to find publications, there is a high chance of rejecting publications with small typos.

The manual evaluation was performed by five researchers only. For each cluster, 10 datasets were recommended, and each researcher has to evaluate an average of 40 datasets. It was a time-consuming task for evaluators to check each of the recommended datasets. For manual evaluation, we required the human judges with expertise on the GEO datasets, which was challenging to find. Further research will entail the scaling of this evaluation process.

### GETc Platform

We developed the GETc research platform that recommends datasets to researchers using the proposed methods. A researcher needs to provide his/her name (as in PubMed) and CV (or list of publications) in the website. After processing his/her publications collected from PubMed, the recommendation system recommends datasets from GEO. Researchers can provide feedback for the datasets recommended by our system based on the evaluation criteria mentioned in Section 3.5. A screenshot of the dataset recommendation system is shown in Figure 5. This platform also recommends datasets using texts/documents, where cosine similarity of text and datasets are calculated, and datasets with a high score are recommended to users. Apart from dataset recommendation, it can also recommend literature and collaborators for each dataset. The platform analyzes time-course datasets using a specialized analysis pipeline (http://genestudy.org/pipeline) (25). We believe that these functions implemented in the GETc platform will significantly improve the reusability of datasets.

## Conclusion and future work

This work is the first step toward developing a dataset recommendation tool to connect researchers to relevant datasets they may not otherwise be aware of. The maximum NDGC@10, P@10 (P) and P@10 (S) of 0.89, 0.78 and 0.61 were achieved based on the proposed method (MIDR) using five evaluators. This recommendation system will hopefully lead to greater biomedical data reuse and improved scientific productivity. Similar dataset recommendation can be developed for different datasets from both biomedical and other domains.

The next goal is to identify the clusters which are not related to datasets and used for recommendations in the present article. These clusters can be removed from further experiments. Later, we plan to implement other embedding methods and test the dataset recommendation system on a vast number of users. A user-specific feedback-based system can be developed to remove datasets from the recommendations. Several additional dataset repositories can be added in the future. Other APIs can also be added to retrieve more complete representation of researcher’s publication history.

**Availability:**  http://genestudy.org/recommends/#/
